# A computational model for regulation of nanoscale glucan exposure in *Candida albicans*

**DOI:** 10.1371/journal.pone.0188599

**Published:** 2017-12-12

**Authors:** Michael J. Wester, Jia Lin, Aaron K. Neumann

**Affiliations:** 1 Department of Mathematics and Statistics and Center for Spatiotemporal Modeling of Cell Signaling, University of New Mexico, Albuquerque, New Mexico, United States of America; 2 Department of Pathology, University of New Mexico, Albuquerque, New Mexico, United States of America; Institute of Microbiology, SWITZERLAND

## Abstract

*Candida albicans* is a virulent human opportunistic pathogen. It evades innate immune surveillance by masking an immunogenic cell wall polysaccharide, β-glucan, from recognition by the immunoreceptor Dectin-1. Glucan unmasking by the antifungal drug caspofungin leads to changes in the nanostructure of glucan exposure accessible to Dectin-1. The physical mechanism that regulates glucan exposure is poorly understood, but it controls the nanobiology of fungal pathogen recognition. We created computational models to simulate hypothetical physical processes of unmasking glucan in a biologically realistic distribution of cell wall glucan fibrils. We tested the predicted glucan exposure nanostructural features arising from these models against experimentally measured values. A completely spatially random unmasking process, reflective of random environmental damage to the cell wall, cannot account for experimental observations of glucan unmasking. However, the introduction of partially edge biased unmasking processes, consistent with an unmasking contribution from active, local remodeling at glucan exposure sites, produces markedly more accurate predictions of experimentally observed glucan nanoexposures in untreated and caspofungin-treated yeast. These findings suggest a model of glucan unmasking wherein cell wall remodeling processes in the local nanoscale neighborhood of glucan exposure sites are an important contributor to the physical process of drug-induced glucan unmasking in *C*. *albicans*.

## Introduction

*Candida albicans* is a normal commensal fungus living on various human mucosal surfaces. It is also an opportunistic pathogen that can cause superficial or systemic infections. Healthcare costs associated with treatment of candidiasis exceed $1 billion annually in the US alone [1, 2]. Innate immune host defense is critical to combating candidiasis, so understanding the host and pathogen factors that impact fungal recognition is both scientifically and clinically significant.

Initial contact between *Candida* and an innate immune cell occurs at the cell wall surface. Because of this, surface accessible cell wall components play an important role in host-pathogen interactions. The *Candida* cell wall contains the polysaccharides chitin, β-(1,3:1,6)-D-glucan and N- and O-linked mannans. Innate immune cells (e.g., dendritic cells, macrophages and neutrophils) sense fungal cell wall polysaccharides via Pattern Recognition Receptors (PRRs), such as the transmembrane C-type lectins (CTLs) DC-SIGN and Dectin-1 that bind mannan and β-(1,3)-D-glucan, respectively. Recognition of β-glucan by Dectin-1 provides powerful immunogenic signals capable of driving fungal phagocytosis and downstream cellular activation in these cell types [3–5]. Activation by PRRs allows leukocytes to fulfill critical roles in innate and adaptive immunity as sentinels of pathogen entry and as antigen-presenting cells for stimulating T lymphocytes [6]. Therefore, regulation of the amount and exposure of immunogenic cell wall ligands is an important facet of the host-pathogen interaction that is the subject of active investigation.

While mannan is abundantly exposed at the surface of *C*. *albicans* cell walls, recent studies have shown that surface exposure of β-glucan is limited to punctate exposures of nanometric dimensions in resting *C*. *albicans* yeast lateral cell walls [7, 8]. However, conditions such as treatment with the antimycotic drug caspofungin, hyphal germination and neutrophilic attack have been reported to cause a phenotype of increased β-glucan exposure in *C*. *albicans* cell walls as observed by conventional, diffraction-limited imaging and dSTORM imaging [8–10]. Caspofungin impairs β-glucan biosynthesis. It is unclear exactly how caspofungin treatment leads to glucan unmasking, but caspofungin's effects on glucan biosynthesis may result in increasing exposure of existing glucan due to normal cell wall mannoprotein turnover and/or cell wall remodeling processes combined with a failure to synthesize new glucan-based cell wall structures. It is consistent with this concept that perturbation of genes related to cell wall biosynthetic processes can also elevate glucan exposure. For instance, deletion of *C*. *albicans* genes directly involved in β-1,3-glucan remodeling (*PHR2*), β-1,6-glucan synthesis (*KRE5*) and global transcriptional regulation of cell wall biosynthetic machinery (*SSN8*) results in increased exposure of β-glucan [9]. Because, β-glucan is the point of attachment for many cell wall mannoproteins, perturbations to glucan biosynthesis and remodeling are likely to adversely impact proper localization of mannan in the cell wall. Moreover, ablation of Mnn2 mannosyltransferase family members diminishes the size and structural complexity of *N*-mannan, in turn reducing β-glucan masking on cell walls [11]. These and other studies [12] support a model wherein N-mannosylated cell wall proteins provide the moiety that masks exposure of β-glucan, allowing *C*. *albicans* to evade innate immunity by restricting Dectin-1's access to β-glucan.

Our super resolution imaging of fine-scale β-glucan exposure geometries has revealed that the majority of glucan exposure sites on *C*. *albicans* yeast lateral cell walls are single glucan/Dectin-1 interaction sites. After caspofungin-mediated unmasking, the cell wall surface exhibits increases in exposure site density, including an increasing number and size of glucan exposures represented by clusters of Dectin-1 binding sites having a median radius of 19 nm [8]. While it is unknown exactly what processes control the local, nanometric-scale exposure characteristics of β-glucan, we hypothesize that passive turnover of cell wall mannoproteins due to degradation and environmental damage, and perhaps also local, active enzymatic remodeling of the cell wall, may account for the changes in β-glucan nanoexposure geometry that we have observed. In the present work, we use a computational approach to test these mechanistic hypotheses by determining whether simulations of a spatially random unmasking process (i.e., passive turnover), alone and with additional locally biased unmasking (i.e., active remodeling) of glucan, can recapitulate the nanoscale glucan exposure geometries that we have observed on masked and unmasked *C*. *albicans* yeast. We conclude that a completely spatially random unmasking process, such as would be expected for passive environmental damage to the cell wall, cannot account for observed nanoscale glucan exposure features. However, models incorporating an additional component of locally biased unmasking provide predictions of glucan nanoexposure geometry that are much closer to observed glucan exposure geometry as measured by super resolution microscopy. This finding is consistent with a composite mechanism of glucan unmasking that involves spatially random unmasking together with a moderate contribution of active local remodeling of cell wall structure.

## Results

Our previous study indicated that the majority of glucan exposed on resting *C*. *albicans* cell walls is present in single Dectin-1 binding sites, termed “singlet glucan exposures”. After caspofungin treatment, higher density of Dectin-1 binding glucan exposure sites appears. Additionally, after drug treatment, glucan exposure sites able to bind multiple Dectin-1 probes, termed “multiglucan exposures”, were increased in density and size. To facilitate comparison of modeling data from this work with experimental data previously described, we present Table 1 that summarizes key experimental results from Lin, *et al* concerning glucan exposure nanostructure in *C*. *albicans* [8]. These data were derived from direct Stochastic Optical Reconstruction Microscopy on fungal cell walls. Measured quantities included the *localization density* (relates to the density of probes attached to glucan on the sample surface), the *singlet glucan exposure fraction* (proportion of glucan exposures representing single ligand/probe interaction sites), the *equivalent radii of multi-exposures* (radial size of clustered glucan exposures), and the *glucan site exposure densities* (number of glucan exposure sites, including both singlet glucan and multiglucan exposures, per unit area). All these quantities were assessed for untreated and caspofungin treated yeasts. Table 1 summarizes the values for the 25th, 50th (median) and 75th percentiles of the two yeast conditions for each quantity of interest used in our study, as specified in the two leftmost columns.

**Table 1:** The values for the 25^th^, 50^th^ (median) and 75^th^ percentile of the experimental data as presented in Lin et al.^8^.

		25^th^ percentile	50^th^ percentile	75^th^ percentile
**% glucan surface area**	Yctrl	0.03	0.04	0.05
	Ycasp	0.19	0.22	0.24
**fraction of singlets**	Yctrl	0.87	0.92	1.00
	Ycasp	0.82	0.85	0.88
**radius of multi-exposures** **(nm)**	Yctrl	5.8	8.6	14.2
	Ycasp	16.2	19.2	23.9
**glucan exposure density** **(number of glucan exposures/μm**^**2**^**)**	Yctrl	5.2	7.0	7.5
	Ycasp	24.6	28.5	34.0

Referring to figures in Lin et al.: Fig 7C (localization density converted to percent glucan surface area observed), Fig 7D (fraction of singlet glucan exposure sites), Fig 8B (equivalent radii of circular exposures representing multiglucan exposure sites), and Fig 8A (glucan exposure density). These values were used to compare with the simulation outputs. Yctrl and Ycasp refer to untreated control and caspofungin treated yeasts, respectively.

**Fig 1:**
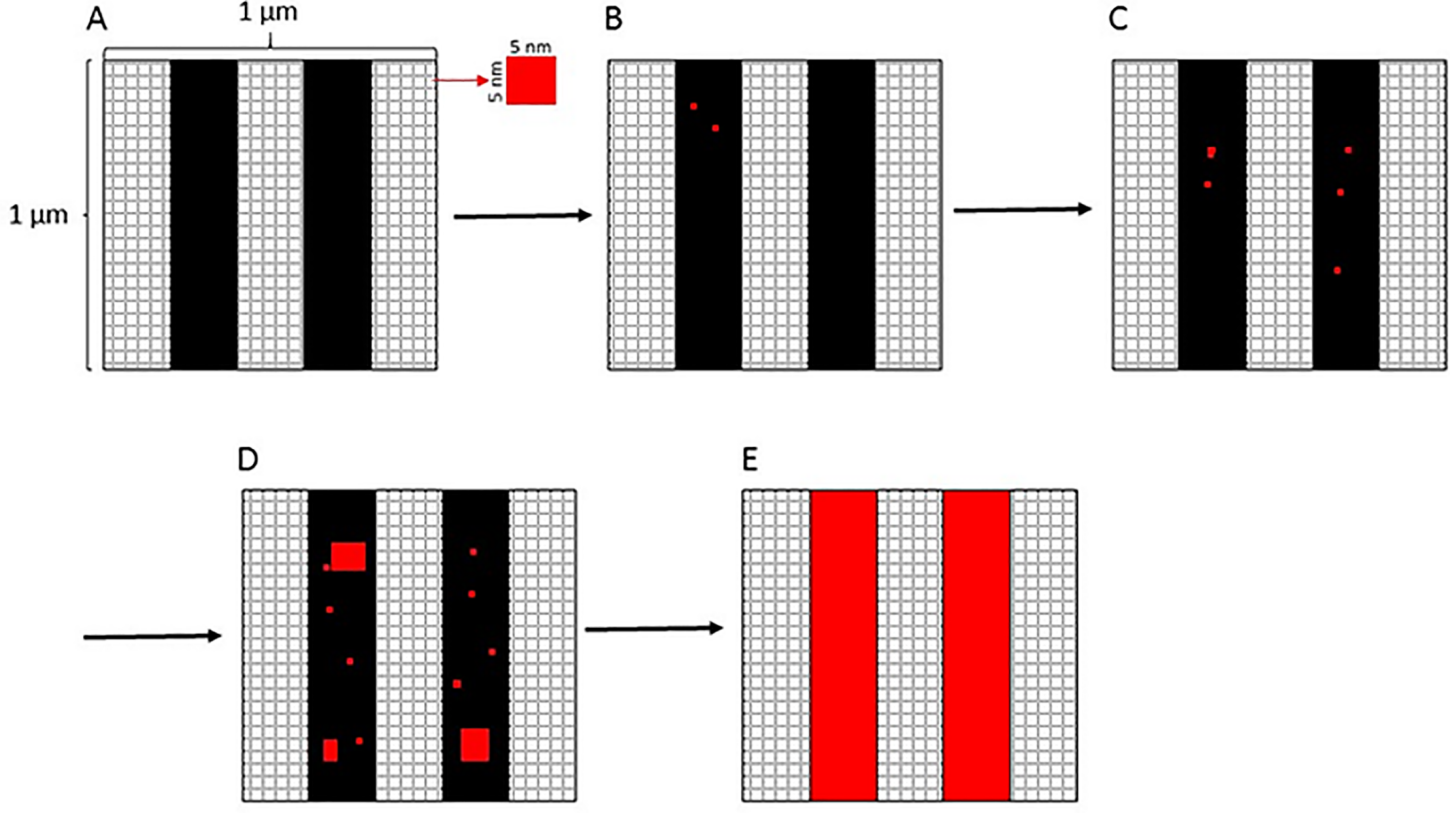
Schematic description of the glucan unmasking model. (A) Masked glucan on *C*. *albicans* cell walls was represented as stripes (black) of a specified width in pixels and covering a specified fraction of the total simulation area (white grid space). The stripes model the presence of fibrillar structures of insoluble glucan in the *C*. *albicans* cell wall. Each pixel represents a 5 nm x 5 nm area in the total square simulation space of 1 um^2^. The glucan unmasking model proceeds by selecting a single pixel at each iteration (black masked glucan or white glucan free) and changing its state. As pixels representing glucan are unmasked, they change their color to red. (B-D) Pixels are chosen for glucan unmasking at each iteration of the simulation, either in a random (P_edge_ = 0) or an edge biased (P_edge_>0) manner. If P_edge_>0, then P_edge_ determines the probability that a pixel will be chosen from the boundary pixels surrounding existing exposures as opposed to a pixel chosen randomly from the surface. (E) The simulation space is 200 x 200 pixels, so after 40,000 iterations all pixels have been selected and all masked glucan has been exposed.

To understand mechanisms that might lead to glucan unmasking, we developed a sequence of iterative computer simulations. Our basic premise was that we could use a simulation space with a grid of regular spatial elements (pixels) to model the region of interest (ROI) analysis results from the pixelated rendering of super resolution microscopy experimental data [8]. In the experimental datasets, each pixel represented a 5 nm x 5 nm region of the sample. This value was derived from an estimate of the largest dimensions of a single Dectin-1 carbohydrate recognition domain (PDB: 2BPD) [13], due to the fact that Dectin-1 was used as the probe for glucan exposure in the experimental studies. Because the ROIs from our experimental data represented a total 1 μm x 1 μm physical space in the specimen, our corresponding simulation was comprised of a 200 x 200 grid of model pixels, each pixel representing a 5 nm x 5 nm area (Fig 1A).

Glucan is not distributed uniformly in fungal cell walls but rather exists in fibril structures and thus can only be unmasked in certain areas [14]. In order for the simulations to reflect this anisotropic distribution of cell wall glucan, we introduced striped regions in the simulation space in which masked glucan was assumed present (black), while glucan was absent from all other areas (white) (Fig 1A). The width of the stripes and the total masked glucan area (expressed as a percentage of the total simulation space) were thus parameters of the simulations. Our simulations were restricted to conditions with uniform stripe width.

**Fig 2:**
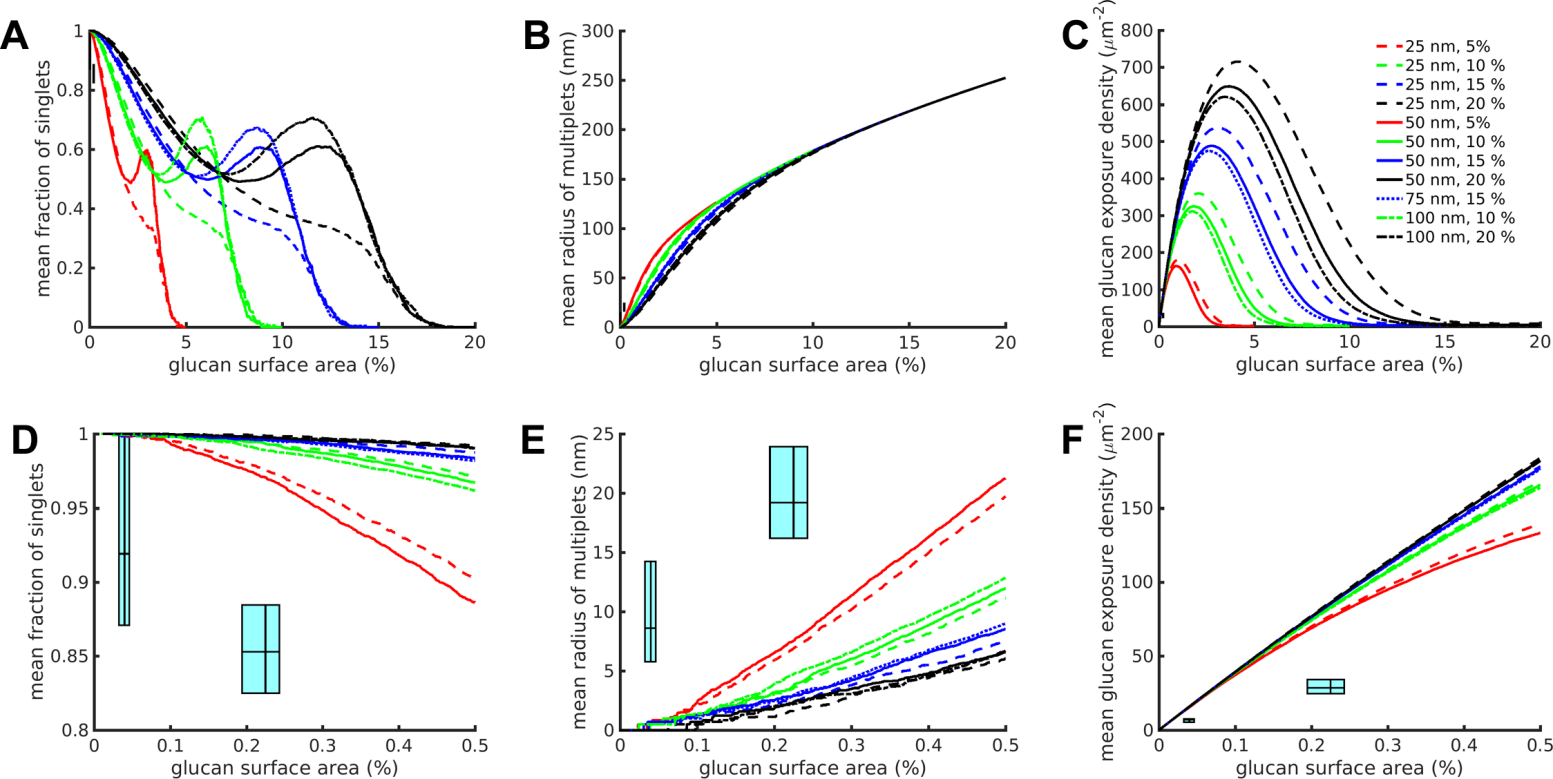
Random unmasking model simulation results did not match the experimental results. (A-C) Full results over the entire simulation for various combinations of stripe width (nm) and glucan coverage (percent) of the simulation space for the three simulation and experimental outputs measured. The lines represent means calculated from n = 100 runs for each condition. (D-F) Zoomed in results corresponding to the experimental data domain. The cyan range boxes express ranges in the style of box plots with the lines representing the 25^th^, 50^th^ (median) and 75^th^ percentiles of the experimental data, going from left to right or bottom to top, on the corresponding axes (percentiles refer to percent glucan surface area on the horizontal axes, and to singlet fraction, equivalent multi-exposure radius or glucan exposure density as denoted on the vertical axes). Within each plot, the left-most cyan range box corresponds to untreated yeast, the right-most to caspofungin-treated yeast.

Results from the computational model of glucan exposure assuming a spatially random unmasking process are illustrated in Fig 2. To compare the results of the simulations of glucan unmasking with the analysis of the experimental data, the singlet exposure fractions, equivalent radii of multiglucan exposures, and glucan site exposure densities from the simulations were plotted versus the simulated percent glucan surface area. This latter quantity is defined as the fraction of exposed pixels per total number of pixels in the simulation space (40,000). It is directly comparable with the experimentally observed localization densities expressed as total exposed glucan area (nm^2^, with each localized glucan probe assumed to occupy 25 nm^2^) divided by the Region of Interest (ROI) area (1 μm^2^ = 10^6^ nm^2^). This means that the percent glucan surface area ranges from the experimental data can be plotted along the horizontal axis of the simulation results. Correspondingly, the ranges of the experimental singlet fractions, equivalent radii of multiglucan exposures, and glucan site exposure densities can be plotted along the vertical axes of the equivalent simulation results. Together, these axial ranges create range boxes of experimental data overlaid on top of the simulation results (Fig 2D–2F, cyan boxes). These boxes express ranges in the style of box plots, representing the 25^th^, 50^th^ and 75^th^ percentiles of the relevant values (percent glucan surface area, singlet fractions, equivalent radii of multiglucan exposures, and glucan site exposure densities). The left and right edges of the boxes represent the 25^th^ and 75^th^ percentiles of percent glucan exposure area in the experimental dataset, with the 50^th^ percentile represented by the vertical line within the box. The bottom and top edges of the boxes represent the 25^th^ and 75^th^ percentiles of experimental dataset measurement as noted on the plot's vertical axis (singlet fractions, equivalent radii of multiglucan exposures, or glucan site exposure densities), with the 50^th^ percentile represented by the horizontal line within the box.

As a concrete illustration of the concepts introduced above, once again consider Fig 1. Panel A represents the initial configuration in which the glucan localization density is zero, that is, there are no exposed pixels. In Panel B, two pixels (in red) have been exposed on the black stripes which represent glucan fibrils. The localization density is therefore 2/40,000, corresponding to 0.005% glucan surface area. The two exposed pixels are isolated from each other, so are both singlets. Since only singlets are present, the singlet fraction is one. In Panels C and D, more pixels are exposed, sometimes as isolated singlets and sometimes collected together into groupings. Each singlet or grouping is a glucan exposure *site*. The localization density is defined as the total number of exposed pixels per unit area, while the glucan site exposure density is the total number of glucan sites per unit area. Biologically, we are comparing the density of probes attached to glucan versus the density of exposures. The singlet fraction is now < 1 as some of the exposed sites are not singlets, this fraction being the number of singlet sites per total number of sites. Finally, for those sites containing three or more pixels, we compute the exposure site area (corresponding to the area bounded by three or more localizations in the experimental data). Rather than compare areas directly, we compute the radii of circles with equivalent areas, as the values have a more understandable interpretation. In Panel E, all the masked glucan has been exposed and is collected together into a single exposure site per stripe, corresponding to a glucan site exposure density of 2/μm^2^ since the ROI area is 1 μm^2^. The localization density and equivalent radii of multi-exposures is at a maximum, while the singlet fraction is now zero. From the data in Table 1, the maximum 75^th^ percentile localization density corresponds to about 100 exposures / μm^2^, which equates to approximately 0.25% glucan surface area, establishing the upper end of the percent glucan surface area range that is applicable to reported experimental observations of glucan exposure nanostructure. Mean behavior of all three simulation outputs are shown over the entire range of the simulations through complete unmasking (Fig 2A–2C), but separate plots detail the experimentally comparable percent glucan surface area range of all outputs for easier examination (Fig 2D–2F). We focused on comparing simulation results to experimental results between the 25^th^ and 75^th^ percentiles of percent glucan surface area exposed relevant to the two yeast experimental conditions, untreated and caspofungin-treated.

Glucan in the cell wall is not homogeneously distributed, but rather is present as insoluble fibrils ([14, 15], see Methods). To account for the effects of glucan fibrils, the simulation space was filled with glucan stripes of width 25, 50, 75 or 100 nm (5, 10, 15 or 20 pixels) wherein the glucan stripes covered 5%, 10%, 15% or 20% of the total simulation space. While stripes are a simplified representation of glucan fibril structure, these width values are based on available experimental evidence (see Methods). We present in Fig 2 the mean output of simulations at various model parameterizations (n = 100 simulations/parameter set). As expected, the percent glucan coverage of the simulation space determines the maximum percent glucan surface area that can be achieved in the simulation (note the final values on the horizontal axis) (Fig 2A–2C). Furthermore, differences in the stripe width change the behavior of simulation output curves, which is especially noticeable on the singlet fraction curves and glucan exposure density as the characteristic shape of the curves is compressed into smaller ranges of percent glucan surface area as the stripe width decreases (Fig 2A and 2C).

**Fig 3:**
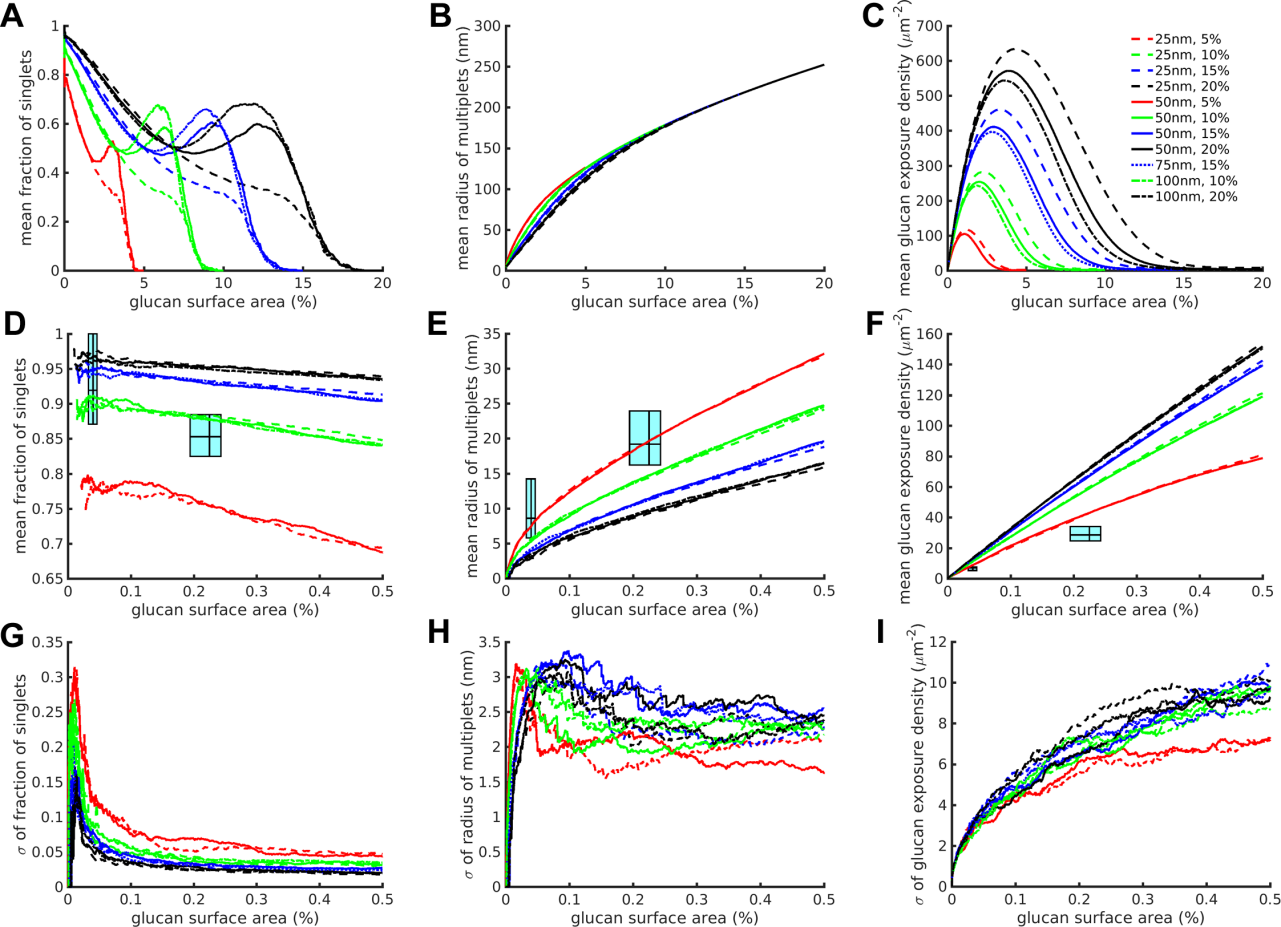
The edge biased unmasking model improves correspondence of simulated and experimental datasets. We show P_edge_ = 4% bias runs as an example. (A-C) Full results over the entire simulation for various combinations of stripe width and glucan coverage for the three simulation and experimental outputs measured. (D-F) Similar to A-C, but showing zoomed in results corresponding to the experimental data domain. The cyan range boxes express ranges in the style of box plots with the lines representing the 25^th^, 50^th^ and 75^th^ percentiles of the experimental data, going from left to right or bottom to top, on the corresponding axes. Within each plot, the left-most cyan range box corresponds to untreated yeast, the right-most to caspofungin-treated yeast. (G-I) The standard deviations of the zoomed in results from D-F. The lines represent mean (A-F) or standard deviation (G-I) values calculated from n = 100 runs for each condition.

Plots in Figs 2D–2F and 3D–3F include experimental data indicated by cyan range boxes: untreated (left-most box in the plot) or caspofungin-treated (right-most box in the plot). If a given model and parameterization is an accurate representation of the process of unmasking, then we expected to find at least some simulation parameterizations that accurately predicted glucan exposure nanostructure, resulting in simulation outputs that would lie within the boxes defined by experimental measurements. The completely spatially random unmasking model predicted glucan nanoexposure characteristics that were inconsistent with experimental observations (Fig 2D–2F). Throughout the realistic range of model parameterizations used, this model overestimated the fraction of singlet exposures and glucan exposure density while underestimating the size of multiglucan exposures.

Because glucan exposure nanostructure cannot be explained by a completely spatially random unmasking process, we modified the model to account for additional mechanisms of glucan exposure. Fungal cell walls are sensitive and malleable organelles capable of rapid and active enzymatic remodeling during cytokinesis and in response to environmental stress [16, 17]. Therefore, we proposed that glucan unmasking might be partially explained by a locally active enzymatic process acting to enlarge existing sites of glucan nanoexposure. We next modified our model to account for such a mechanism of partially edge biased unmasking, where the degree of bias toward unmasking at the edge of an existing exposure (versus unmasking at a new site randomly located on the cell wall surface) is defined by the new parameter P_edge_ (see Fig 1). P_edge_ is the probability that in a masked glucan region a boundary pixel surrounding an existing exposure was chosen rather than a completely random pixel. In other words, *edge biased unmasking* chooses pixels at the edges of existing exposures thus expands existing glucan exposure sites. P_edge,_ satisfying 0 ≤ P_edge_ ≤ 1, is therefore the probability that an edge pixel is chosen at each iteration, the rest of the time pixels are randomly chosen somewhere on the simulated surface. In this scenario, the random unmasking model described previously is simply the situation when P_edge_ = 0.

We performed the same sort of analysis with the edge biased unmasking model as we did with the spatially random unmasking model, running simulations on a range of P_edge_ from 0.01 to 0.95. An example for P_edge_ = 0.04 (4% bias) is given in Fig 3, with simulation outputs for the complete range of glucan unmasking conditions (Fig 3A–3C) and a detailed range of percent glucan surface area corresponding to comparative experimental data (Fig 3D–3F), similarly to Fig 2. In general, results from the edge biased model with P_edge_ = 0.04 predict glucan exposure nanostructural features that are much closer to experimentally measured glucan exposures than the spatially random model, as shown by their closer approach to experimental data boxes. Across the range of edge biased unmasking model simulations with P_edge_ = 0.04, the variance of the three model outputs was consistent as shown by the plot of standard deviations at all stripe width and percent total glucan parameterizations (n = 100 replicate simulations/parameterization) (Fig 3G–3I).

To determine an optimum value for P_edge_, we defined a metric to compare the simulated mean curves at the experimental percent glucan surface area medians (untreated and treated) with the corresponding experimental medians for each of the three variables examined (singlet fraction, equivalent radii of multiglucan exposures, and glucan exposure density; see Table 1). For each of the three variables, y, we first computed a normalized signed discrepancy,

$$D_{y}=\frac{y_{experimental}-y_{simulated}}{y_{experimental}}$$

then computed the sum of the absolute values,

$$D=|D_{singletfract}|+|D_{radii}|+|D_{density}|$$

to yield a total discrepancy for either untreated or treated experimental results. D is a weighted sum of the | D_y_ |'s, where all the weights have been chosen to be one, not favoring any of the three variables in the sum. We considered that the value of P_edge_ that produced curves with the lowest total discrepancy to be defined as the simulation parameterization giving optimum predictions of experimentally observed glucan exposure nanostructure.

**Fig 4:**
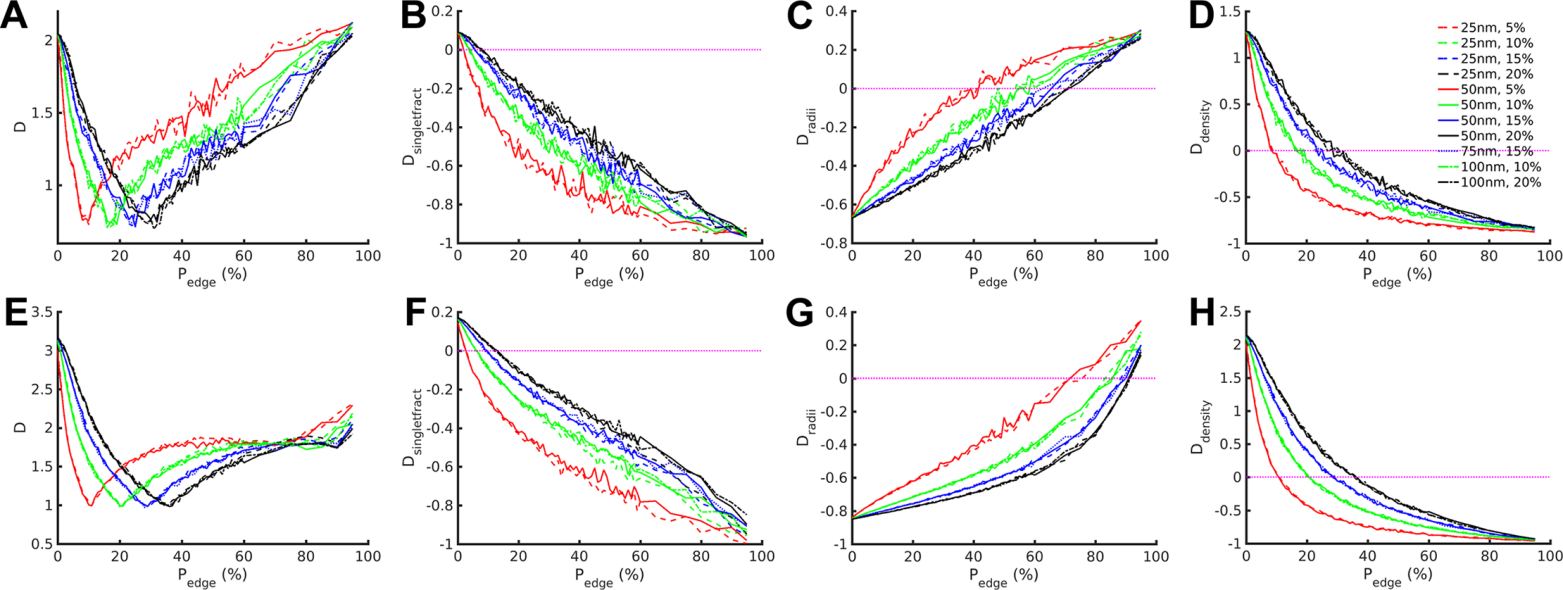
Model/Experiment discrepancy calculations quantify model performance and identify model parameterizations with optimum fit to experimental data. To describe the goodness of fit of the models, we measured discrepancies of various simulation outputs as a function of P_edge_. Data are displayed for untreated (A-D) and caspofungin-treated (E-H) yeasts. Note that P_edge_ = 0 corresponds to the random unmasking model. For each series, results are given for the total discrepancy (A,E) and the individual signed contributions of the three variables of interest [singlet fraction (B,F), equivalent radii of multi-exposures (C,G), and glucan exposure density (D,H)]. Dotted magenta lines in B-D and F-H denote the zero value for the indicated discrepancy metric, which is the value of optimum fit of model results to experimental results for an individual output. The above mean values are calculated from n = 100 runs for each condition.

In Fig 4, we plot D and its three contributing normalized signed discrepancies (D_singletfract_, D_radii_ and D_density_) as a function of P_edge_ for the untreated (Fig 4A–4D) and caspofungin treated (Fig 4E–4H) yeasts. A discrepancy of zero or values near zero is desirable. In all cases, the normalized signed discrepancies, D_y_, approach zero as P_edge_ increases up to some limit that depends on the output quantity (y), treated condition, percent glucan coverage and stripe width (Fig 4B–4D and 4F–4H). The total discrepancy, D, which combines the | D_y_ |’s, also approaches zero over intervals that depend on the treated condition, percent glucan coverage and stripe width (Fig 4A and 4E). In these latter ranges determined by the total discrepancy, the edge biased unmasking model is always an improvement over the random unmasking model because the magnitude of D in these ranges is always less than the magnitude of D at P_edge_ = 0. At values of P_edge_ greater than the occurrence of the minimum D, the total discrepancy begins to rise, indicating a non-optimal fit between the model and experimental data introduced by too much edge bias. However, the edge biased unmasking model (all P_edge_>0) continues to provide better predictions of experimental data than the spatially random unmasking model (P_edge_ = 0) until extreme values of P_edge_ are reached (i.e., P_edge_ = 0.95 for the untreated yeasts; the edge biased unmasking model was always better for caspofungin treated yeasts).

The different stripe widths (depicted as different line styles) have very minor effects on the quality of fit of simulation predictions to experimental measurements for all three measured quantities, while percent of total area containing glucan (depicted as different line colors) had a much stronger effect. We note that for the total discrepancy D (Fig 4A and 4E), the smaller the percentage of the glucan coverage, the steeper the slope of the curves (in absolute value) is at any particular value of P_edge_ ≤ the value of P_edge_ corresponding to the minimum D for the given percentage of glucan coverage. Similar comments can be made for most of the D_y_’s (Fig 4B–4D and 4F–4H). Examination of individual outputs’ signed normalized discrepancies, focusing for illustrative purposes on 5% glucan coverage, shows that singlet fraction discrepancies decrease as P_edge_ rises, transitioning from positive to negative discrepancies (under- to overestimate of experimental values) at P_edge_ = 0.02/0.03 (untreated/caspofungin-treated) (Fig 4B and 4F). Multiglucan equivalent radius discrepancy increases with P_edge_, with negative discrepancies until about P_edge_ = 0.39 (untreated) or P_edge_ = 0.70 (caspofungin-treated) (Fig 4C and 4G). Glucan exposure density signed normalized discrepancy decreases with rising P_edge_, not reaching zero until P_edge_ = 0.09/0.11 (untreated/caspofungin-treated) (Fig 4D and 4H).

All 11 conditions of stripe width and glucan coverage produced the same trends. However, the values of the transitions varied, but always with caspofungin-treated greater than untreated. It is notable from the above observations that the three outputs relating to glucan nanostructure are optimally predicted for different ranges of P_edge_. Therefore, the best fit model parameterizations must always represent a compromise between these three outputs wherein divergence of model predictions from experimental observations is simultaneously minimized, but individual outputs cannot all simultaneously achieve global minimum discrepancies.

Since some of the D_y_ cross zero, | D_y_ | will turn back from zero, which is evident in the plot of D where the glucan coverage curves exhibit minima ranging from P_edge_ = 0.10–0.36 depending on the percent glucan coverage. In general, the larger the percent glucan coverage, the higher the value of P_edge_ becomes where D is minimized. The values of the minimal total discrepancies are similar over all stripe widths and percent glucan coverages, within the untreated or treated groups, respectively. In Table 2, we report the values of singlet fraction, equivalent radii and exposure density predicted by the edge biased unmasking model at the four different percent glucan coverages simulated. These data are shown for the P_edge_ that provided the minimum D, which is considered the optimal model parameterization providing the best fit to experimental data, so as to focus on model parameterizations that optimally fit the experimental results shown in Table 1. Also, the data in Table 2 only concern simulations with 50 nm glucan fibril widths because: 1) our models were quite robust to variations in fibril width over the experimentally relevant range of percent glucan surface area and 2) this fibril width is considered a good representative value for native fungal glucan fibril sizes based on the available literature (see Methods).

**Table 2:** Simulation outputs (mean ± standard deviation) from model parameterizations with optimal fits to experimental results.

% Glucan Coverage	Min DP_edge_	Singlet Fraction	Equivalent Radius (nm)	Exposure Site Density (μm^-2^)
5	10	0.64 ± 0.23	9.4 ± 2.1	6.8 ± 3.1
10	19	0.66 ± 0.21	9.3 ± 1.9	7.1 ± 2.9
15	25	0.66 ± 0.17	9.3 ± 1.5	7.1 ± 3.1
20	32	0.63 ± 0.22	9.5 ± 1.7	6.9 ± 2.8
5	11	0.63 ± 0.09	24.0 ± 1.5	27.8 ± 5.7
10	21	0.63 ± 0.09	23.9 ± 1.5	28.1 ± 6.4
15	29	0.63 ± 0.09	23.9 ± 1.4	28.2 ± 5.9
20	36	0.63 ± 0.10	23.8 ± 1.2	28.9 ± 6.3

All results shown correspond to simulations conducted at a representative glucan fibril width of 50 nm, and at values of P_edge_ associated with the minimum D for the given percent glucan coverage and treated condition. The top half of the table is untreated results, while the bottom half is caspofungin-treated results.

The best predictions of experimental data by the model are only achieved when it is assumed that a larger degree of edge-biased unmasking (larger P_edge_) happens in cells treated with caspofungin. This finding is consistent with the fact that caspofungin represents a cell wall stressor, so heightened activity of local cell wall remodeling enzymes is expected to be present in *C*. *albicans* yeast treated with this drug. The fact that this prediction emerges from our model lends confidence that the glucan exposure regulatory processes embodied in the model do bear close relation to mechanisms controlling glucan exposure in *C*. *albicans* yeast cells.

**Fig 5:**
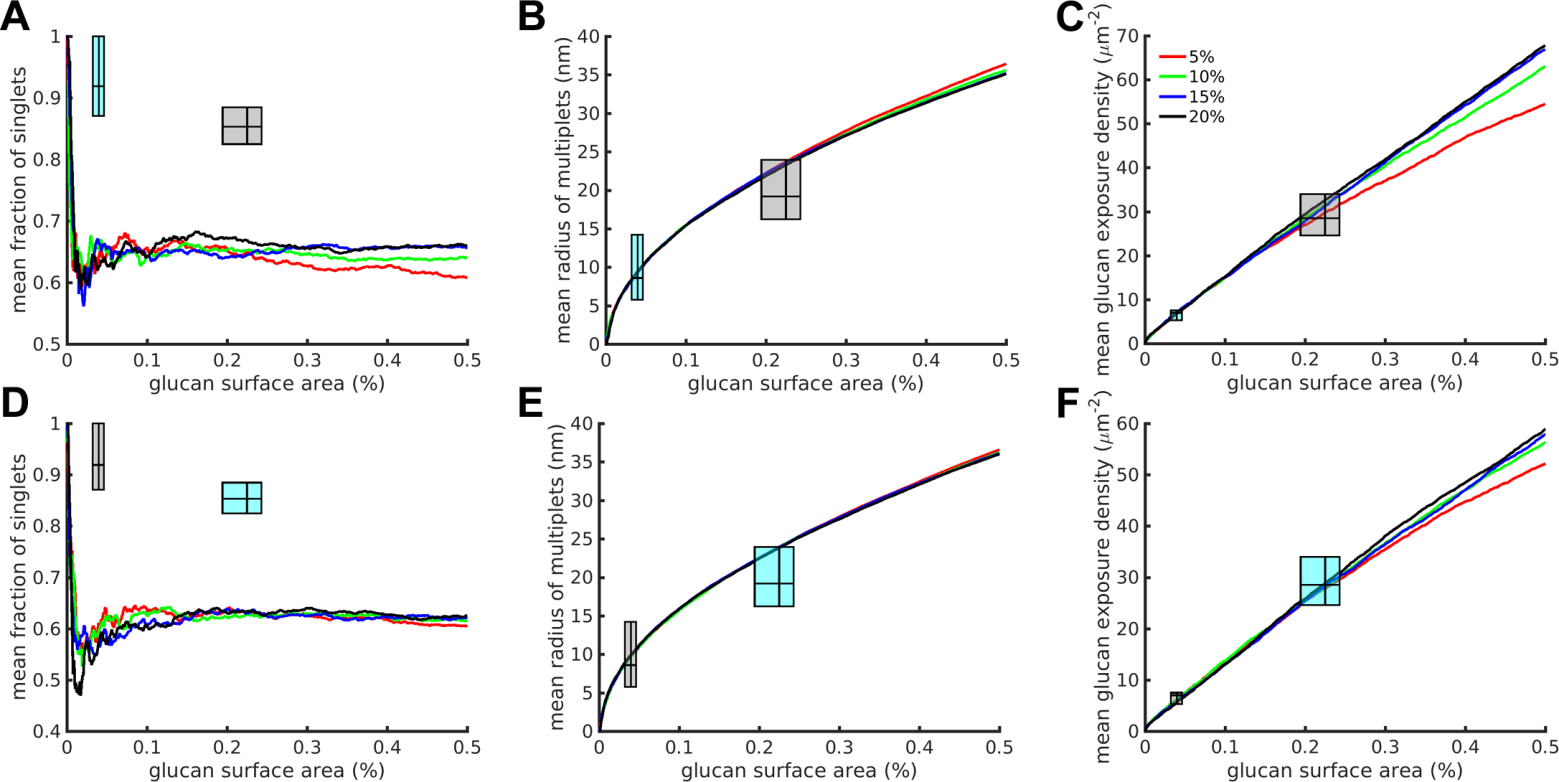
Edge-biased unmasking model parameterizations with optimum global performance predict experimental outputs significantly better than random unmasking models. We compared the simulated to the experimental results using the P_edge_’s that produced the smallest total discrepancies (minimum D) for 5, 10, 15 and 20% glucan coverage with fibril width 50nm in untreated and treated conditions. The actual P_edge_ values used in the depicted model results are given in Table 2. Data are displayed for untreated (A-C) and caspofungin-treated (D-F) yeasts, corresponding to singlet fraction (A,D), equivalent radii of multi-exposures (B,E), and glucan exposure density (C,F). The range boxes express ranges in the style of box plots with the lines representing the 25^th^, 50^th^ and 75^th^ percentiles of the experimental data, going from left to right or bottom to top, on the corresponding axes. The left-most range box corresponds to untreated yeast, the right-most to caspofungin-treated yeast. Within each plot, the above values are calculated from n = 100 runs for each condition. For emphasis, the range boxes representing untreated experimental data are colored cyan in A-C, which concern simulations of untreated yeast. Conversely, the range boxes representing caspofungin-treated experimental data are colored cyan in D-F, which concern simulations of caspofungin-treated yeast. In every case, range boxes for the sample type less relevant to a given plot are de-emphasized by grey coloration, but are retained for reference.

To provide a more visual comparison of the optimal predictions of the edge biased model, we focused on the P_edge_ parameterizations that provided the optimal fits to the experimental data for each of the four different percent glucan coverages tested for untreated and treated conditions (Fig 5). These curves represent the best performance of the model, that is, the simulated results taken at the P_edge_ corresponding to the minimum D for each individual percent glucan coverage for a representative fibril width of 50 nm (see also Table 2). This allows the predictions of all three outputs (singlet fraction, equivalent radius of multi-exposures, and exposure site density) to be individually assessed against experimental values. Under optimal P_edge_ parameterization (i.e., the P_edge_ at which D is minimized for a given parameterization of stripe width and glucan coverage), our model predicts mean glucan exposure site density quite well for untreated and caspofungin-treated cases (Fig 5C and 5F). Similarly, the model provides good predictions of mean equivalent multi-exposure radii for both untreated and treated cases (Fig 5B and 5E). While the consideration of non-zero edge biased unmasking probabilities in our model did clearly improve predictions of mean singlet fraction, we find that optimal model parameterizations did not provide predictions that fit experimental data for singlet fraction as well as the other outputs, for either untreated or treated conditions (Fig 5A and 5D).

## Discussion

Genetic and biochemical approaches have advanced our conceptual understanding of glucan masking and its impact on host-pathogen interactions [9, 10, 18]. However, the field still lacks physically detailed and quantitatively testable mechanistic models of how glucan masking and unmasking unfolds in *C*. *albicans* at the fine spatial scales relevant to engagement of Dectin-1. Here, we used mathematical modeling to simulate physical processes regulating glucan exposure during glucan unmasking by the antimicrobial drug, caspofungin, and tested the model predictions against super resolution imaging observations of glucan exposure under the same conditions. We first considered a totally random unmasking process. Glucan exposure nanostructure predicted by this mechanism was not well matched to experimental observations, excluding a fully random process as an adequate mechanistic description of glucan exposure. We then introduced P_edge_, a parameter indicating the extent of local glucan unmasking near sites of existing glucan exposure (e.g., glucan remodeling enzyme activity) that remodel the cell wall dynamically to alter glucan exposure. Simulations of glucan exposure induced by caspofungin according to this model improved accuracy of predictions of experimentally observable glucan exposure nanostructure. Furthermore, the model predicts that a greater degree of bias toward local glucan unmasking activity (P_edge_) is necessary to optimally predict glucan exposure nanostructure in caspofungin treated yeasts, while control untreated yeasts achieve optimum results at lower P_edge_. Because caspofungin inhibits the fungal β-(1,3)-glucan synthase, it represents a significant cell wall stressor. Under conditions of cell wall stress, *C*. *albicans* activates cell wall repair pathways, leading to increased enzymatic remodeling of the cell wall. So, the prediction of increased local glucan unmasking that emerged from our study is consistent with expected cell wall remodeling activities during drug treatment.

Prior reports have implicated mannan biosynthesis and cell wall incorporation as a key control point in regulating glucan exposure. *C*. *albicans* N-linked mannan biosynthesis involves the *C*. *albicans* Mnn2 family of α-1,2-mannosyltransferases [19]. Deletion of CaMNN2 family genes attenuates N-mannan structure and leads to increased glucan exposure [11]. Caspofungin inhibits the activity of the multi-protein β-(1,3)-glucan synthase complex (CaGsl1p, CaGsl2p, CaGsc1p and CaRho1p) [14, 20]. Caspofungin may impair the development of the glucan scaffold to which mannoproteins attach, thus degrading glucan masking in the outer cell wall. Consistent with this, The Ca*kre5* deletion mutant has reduced β-(1,6)-glucan which links mannoproteins to the glucan network; this mutant possesses strongly increased cell wall glucan exposure [9, 21, 22]. Specifically, β-(1,6)-glucans can attach mannoproteins to glucan via a GPI anchor remnant [23, 24] in a process that requires Ca*DFG5* and Ca*DCW1* [25, 26]. In *S*. *cerevisiae*, ScCdc1p regulates the efficiency of GPI-anchored protein transfer [27]. So, glucan exposure may also be impacted by the ability to incorporate GPI-linked cell wall proteins. However, it is important to recognize that impaired cell wall polysaccharide biosynthesis or incorporation via chemical or genetic perturbations may cause numerous compensatory changes to cell wall structure and composition that make it difficult to disentangle direct and indirect effects on glucan exposure. It is particularly challenging to know how global perturbations of cell wall biosynthesis may be able to directly impact the local (nanoscale) pattern of glucan exposure that was particularly important in our model.

Because our model stresses the importance of a 10–36% bias toward local cell wall synthesis/remodeling in the development of glucan nanostructure, enzymes that are present in the outer cell wall and capable of locally modifying cell wall structure to impact glucan exposure are of particular relevance. Such systems include glucosidases that cleave glycosidic bonds in glucan or glucosyltransferases that mediate attachment of oligosaccharide chains to glucans. These cell wall enzymes can be divided into three classes: 1) carbohydrate-active enzymes found in the outer cell wall but not known to be covalently attached, 2) similar enzymes covalently anchored to the glucan scaffold, and 3) proteolytic enzymes anchored in the cell wall. First, proteomic studies have identified CaBgl2p (β-(1,3)-glucosyltransferase), CaXog1p (exo-β-(1,3)-glucosidase), and CaEng1p (endo-β-(1,3)-glucosidase) as *C*. *albicans* outer cell associated proteins [28–32]. These secreted enzymes could mediate removal or rearrangement of glucan in the outer cell wall, perhaps by interacting with surface accessible glucan exposures from the cell exterior. Indeed, Garfoot et al. reported a similar mechanism of glucan exposure regulation for the homologous Eng1p in *H*. *capsulatum* [33]. Second, several GPI-anchored cell wall proteins capable of modifying β-glucan have also been identified in the outer cell wall of *C*. *albicans*, including CaExg2p (exo-β-(1,6)-glucosidase), CaPhr1p and CaPhr2p (β-(1,3)-glucosyltransferases), and CaScw1p (glucosidase) [32, 34, 35]. Ca*phr2* deletion mutants display increased glucan exposure [9]. CaScw1p (MP65) is an abundant cell wall mannoprotein that likely provides significant glucan masking function, suggesting that glucan masking moieties in the cell wall may provide not only a passive steric blockade of access to glucan, but they may also have significant catalytic activity that locally shapes cell wall structure [35–37]. Additionally, a similar set of GPI-anchored cell wall enzymes with chitin remodeling activity have also been found in the *C*. *albicans* outer cell wall, including CaCrh11p (a transglycosylase that links glucan to chitin) and CaCht2p (chitinase). We speculate that these enzymes may locally control a degree of chitin/glucan crosslinking in the outer cell wall. Third, the *C*. *albicans* outer cell wall contains CaSap9p and CaSap10p which are GPI-anchored aspartyl proteases [32]. CaSap9p is homologous to the *S*. *cerevisiae* "sheddase" yaspin, ScYps1p, that can cleave and release other cell wall anchored proteins [38]. Indeed, CaSap9p and CaSap10p have known proteolytic activity against a number of the GPI-anchored cell wall proteins [39], suggesting that these proteases may locally regulate the level of other cell wall anchored enzymes present within the neighborhood defined by gyration about their β-(1,6)-glucan tethers. Thus, several systems are known that have the potential to influence cell wall structure through acting interfacially at the cell wall/medium boundary or by virtue of their anchorage in the cell wall by flexible GPI-remnant anchors to β-(1,3)-glucan chains. Further studies are needed to determine if these specific enzymatic systems might provide the locally biased enzymatic activities predicted to be important for determining glucan exposure nanostructure in our computational models.

Dectin-1 activation by fungal β-glucan is a key determinant of host defense against *C*. *albicans* infection [40, 41]. Several lines of evidence suggest that understanding the biophysical nature of Dectin-1/glucan at nanoscale dimensions is significant for antifungal immunity. First, Goodridge et al. stimulated Dectin-1 expressing leukocytes with β-glucan attached to 50, 200 and 500 nm diameter particles, and observed a positive correlation between nanometric length scale of a particular glucan presentation and ROS response [42]. While it is true that particle size might also impact the rate of particle internalization and termination of Dectin-1 signaling [43], these suggest that glucan nanostructure may impact Dectin-1 signaling. Second, Dectin-1 activates an ITAM-like signaling process, engaging Syk via a hemITAM motif with only a single ITAM phosphotyrosine motif [44, 45]. Because a tandem ITAM motif is more typical and supports bivalent interaction with dual SH2 domains of Syk, it is thought that Dectin-1 multimerization is important for Syk recruitment and signaling [46–49]. This is supported by a crystal structure indicating dimerization of ligated Dectin-1 [13]. Third, high-resolution methods are increasingly revealing the importance of nanostructure in cell wall biology. Atomic Force Microscopy (AFM) studies have demonstrated force dependent formation and propagation of CaAls5p adhesin nanodomains [50]. AFM has also been used to characterize glucan exposure and physical properties of cell walls in Ca*cho1* and Ca*kre5* deletion mutants [22]. Our previous studies using dSTORM super resolution imaging have revealed that both glucan exposures in *C*. *albicans* and C-type lectins on dendritic cells exhibit regulated nanoscale organization [8, 51]. For instance, >90% of glucan exposure sites on *C*. *albicans* yeast and hyphal lateral cell walls are restricted to supporting only single Dectin-1 receptor binding, but unmasking glucan with sub-MIC caspofungin increases the density and size of larger glucan exposures capable of supporting multivalent Dectin-1 ligation [8]. Therefore, the size of glucan exposure sites on the cell wall and their valency of interaction with Dectin-1 at nanometric dimensions is relevant to engagement and aggregation of the receptor. It is furthermore likely to be important for understanding Dectin-1 activation. The nanobiology of glucan and Dectin-1 is a gap in knowledge that must be filled if physically realistic and evidence based models of Dectin-1 engagement are to be propagated and tested.

At optimal P_edge_, our model yielded good predictions for experimental values of glucan exposure density and multi-exposure radii, but less accurate predictions of the fraction of singlet exposures. Optimal model performance is a compromise to achieve best results for all three outputs in one model parameterization. Future improvements in model performance may require a more complex model of pools of glucan in the *C*. *albicans* cell wall that can contribute to surface exposure. We have considered glucan fibrils underlying the masking mannan layer to be the sole source of glucan exposure. However, biochemical fractionation of cell wall glucan by alkali solubility identifies two pools of β-(1,3;1,6)-glucan: 1) an alkali-insoluble fraction that is considered to contain the deep cell wall glucan fibrils that we have modeled [15, 52] and 2) an alkali-soluble fraction that is highly branched and has a low degree of polymerization [53, 54]. The alkali-soluble glucan probably resides in the outer cell wall because it is intimately associated with mannan [55–57]. It is possible that these alkali-soluble glucans of the outer cell wall contribute to a population of predominately singlet glucan exposures by virtue of their small size and close association with masking mannans. Future models that consider this additional source of glucan exposure sites might provide predictions of singlet glucan exposure fraction that would more closely match experimental values. Continued efforts to use modeling coupled with experimental observations of glucan nanostructure are likely to provide a fruitful approach to deepening our understanding of the fine structure of the *C*. *albicans* cell wall.

## Methods

### Simulations

Our initial model simulated a completely spatially random glucan unmasking process, consistent with a process driven by random environmental damage to the cell wall and/or cell wall turnover leading to glucan exposure. In this model, a single unique model pixel was converted from the masked to the unmasked state on each iteration (Fig 1B–1E). If the model pixel was within one of the stripes, this conversion represented glucan unmasking, creating a glucan exposure (red). If the model pixel was outside the stripes, no change resulted for glucan exposure. In either case, each individual pixel was converted exactly once, so that the total number of iterations was the same as the total number of pixels (40,000). Also, in random modeling, the unmasking process can occur in any unconverted pixel in the simulated space with equal probability. For this reason, we compared the results of random unmasking anywhere in the simulation space to simulations in which only pixels in the stripes were selected at each iteration. The results were very similar (S1 Fig). This comparison acted as a control, showing that the results obtained from the random unmasking model are not merely an artifact of having partial coverage of glucan in the simulation space.

In the random model, at each iteration, the total number of glucan exposure sites and their areas were tabulated. A glucan exposure site was defined as: 1) an isolated "singlet" exposure consisting of a single exposed pixel or 2) an extended "multiglucan" region consisting of three or more pixels with exposed glucan sharing at least one edge with other members of the region. (Two adjacent pixels, a doublet, were treated in the analysis as two singlets in order to fairly compare with the analysis of the experimental data in which an isolated pair of localizations closer than the clustering radius was treated in a similar manner.) The area of a glucan exposure site was therefore computed simply from the number of pixels that composed it. The total number of glucan exposures at each iteration was then the sum of the number of pixels composing all of the glucan exposure sites. The corresponding area was expressed as the percent glucan surface area exposed in order to provide a common axis to follow the progress of unmasking as variously parameterized simulations were iterated. The total number of glucan exposure sites at the end of the simulation (complete unmasking) was thus the number of stripes and their areas were the stripe areas.

To compare simulation results with experimental data, we computed the localization density (number of glucan exposures per μm^2^), the fraction of singlet glucan exposure sites (isolated unmasked pixels), the equivalent radii of circular exposures representing multiglucan exposure sites (sites with three or more contiguous exposed pixels; nm), and the glucan exposure density (number of glucan exposure sites, including both singlet glucan and multiglucan exposure sites, per μm^2^).

Examining the results from the initial model (see Results), we saw that the experimental data did not match well with the simulations. This led us create a modified model introducing edge biased unmasking and to run a second series of simulations using this model. In the modified model, a third parameter, the edge unmasking probability, P_edge,_ was introduced. At each iteration, a uniform random number, x, between 0 and 1 inclusive, was chosen. If x ≤ P_edge_, a boundary pixel just outside the edge of a pre-existing glucan exposure site (and within a stripe of masked glucan) was chosen, otherwise a random pixel was selected with no restrictions on its location. The boundary pixel chosen was selected randomly from all possible boundary pixels. If there were no pre-existing glucan exposure boundary pixels (for example, at the start of a simulation), then a random pixel was picked. This allowed unmasking activity to be balanced between growth of existing exposure sites (representing local unmasking) during some specified fraction of the time and completely spatially random unmasking (representing random environmental effects).

In the extreme, P_edge_ = 0 corresponds to the random unmasking model, while P_edge_ = 1 causes the first exposure in a stripe to expand to cover the entire stripe before a new exposure in another stripe can occur.

### Implementation of simulations

Simulations and analysis of simulation outputs were implemented in custom MATLAB code, available at http://stmc.health.unm.edu/tools-and-data/ and https://doi.org/10.6084/m9.figshare.5606035. A semi-MATLAB style pseudocode of the unmasking algorithm for P_edge_ ≥ 0 is provided in S2 Fig.

### Parameterization of simulations

Models were parameterized with 5%, 10%, 15% or 20% of the total area of the simulation space as initially masked glucan arranged in vertical stripes of a specified widths of 25, 50, 75, 100 nm (5, 10, 15, 20 pixels). In the edge biased model, simulations were run at P_edge_ values of 0.01 to 0.60 by 0.01 and 0.65 to 0.95 by 0.05.

Stripes of glucan in our simulation space were intended to model the presence of fibrillar structures of insoluble glucan in the *C*. *albicans* cell wall. Osumi measured these fibrils in the cell walls of recovering protoplasts using low-voltage SEM methods [14]. Elemental glucan fibrils were observed to be 1–2 nm wide. However, most glucan fibrils laterally self-assembled into larger bundles of fibrils, and the presence of these larger fibrils increased in more mature cell walls. Fibrils were typically greater than 20 nm wide and ranged up to approximately 200 nm. We chose to simulate a range of fibril widths ranging from 25–100 nm in width, representing the range of fibrils that were most commonly observed. This general size range of fibrils is further supported by the observation of fibrillar structures of approximately 25 nm width in fungal cell walls by AFM [58]. Furthermore, we used *en face* images of glucan fibrils from Osumi's paper to estimate the percent area composed of glucan fibrils. In these images, glucan fibrils are brighter than the background. We thresholded these images at one standard deviation above the mean grey value of the image to objectively identify glucan fibrils in a mask image. The fractional area composed of glucan fibrils so identified was ~10%. Note that because protoplast walls are still growing, this value may be an underestimate of glucan fibril fractional area of the inner cell wall surface for a mature cell wall. To complement this analysis, we similarly analyzed images showing fibrillar inner cell wall structure in *S*. *cerevisiae* from Kopecka, et al [15], and we found that these glucanase-sensitive fibrils covered ~20% of the inner cell wall surface. *S*. *cerevisiae* cell walls are thought to be similar in structure to *C*. *albicans*, and this latter study is likely more indicative of glucan fibrillar structure in a mature Ascomycetous cell wall. Therefore, we simulated a range of percent glucan areas that straddled this range of values, emphasizing slightly higher values in the simulated range upon the assumption that recovering protoplast glucan fibril densities could be somewhat less than a fully matured cell wall.

## Supporting information

S1 Fig Comparison of the random unmasking model (option 2, solid lines) with the control model (option 1, dashed lines). We compared running the random unmasking model in which a random pixel somewhere in the entire simulation space was chosen at each iteration (opt 1) to a control model in which only a random pixel in one of the masked glucan stripes was selected per iteration (opt2) (in the latter case, the number of iterations was adjusted to be just sufficient to flip all the pixels in the given set of stripes). The results for all parameter combinations simulated were nearly identical for the two models for the variables of interest [singlet fraction (A), equivalent radii of multi-exposures (B), and glucan exposure density (C)]. Given that, we chose to simply use the random unmasking model (and the edge biased unmasking model, which was an extension of the random unmasking model) in subsequent studies as it seemed to better mimic the experimental processes we expected might be present. The above values were calculated from n = 100 runs for each condition.
(TIF)

S2 Fig Unmasking algorithm written in semi-MATLAB style pseudocode. Cellwall represents the 200 x 200 pixelated simulation space (which also can be indexed from 1 to 40,000 using one-dimensional indices). Boundary collects the (orthogonal) boundary pixels of existing glucan exposure sites within the masked stripes. Masked keeps track of which pixels are available to be chosen, however, only those in non-fixed regions cover masked glucan and so can be exposed. The actual MATLAB algorithm was written in such a way that it can be run on multiple processors simultaneously.
(DOCX)
