# Supplementary figures and images for: A computational model for regulation of nanoscale glucan exposure in Candida albicans
Source: PLoS One. 2017 Dec 12;12(12):e0188599. doi: 10.1371/journal.pone.0188599 (PMC5726713; doi:10.1371/journal.pone.0188599)

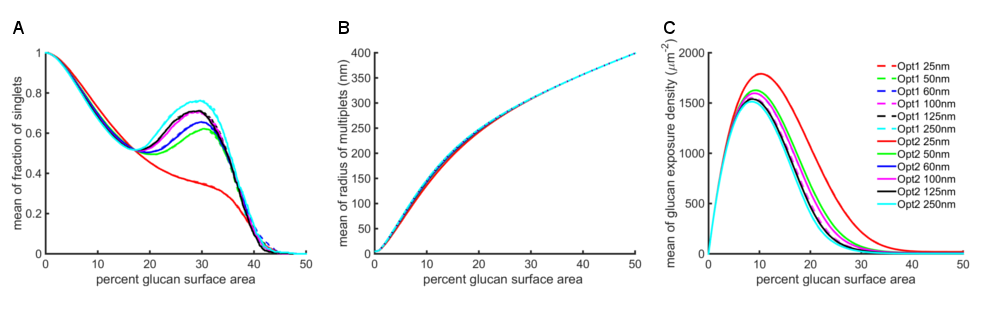

Supplement: S1 Fig — We compared running the random unmasking model in which a random pixel somewhere in the entire simulation space was chosen at each iteration (opt 1) to a control model in which only a random pixel in one of the masked glucan stripes was selected per iteration (opt2) (in the latter case, the number of iterations was adjusted to be just sufficient to flip all the pixels in the given set of stripes). The results for all parameter combinations simulated were nearly identical for the two models for the variables of interest [singlet fraction (A), equivalent radii of multi-exposures (B), and glucan exposure density (C)]. Given that, we chose to simply use the random unmasking model (and the edge biased unmasking model, which was an extension of the random unmasking model) in subsequent studies as it seemed to better mimic the experimental processes we expected might be present. The above values were calculated from n = 100 runs for each condition. (TIF) [file pone.0188599.s001.tif]
