# Supplementary material for: A computational model for regulation of nanoscale glucan exposure in Candida albicans
Source: PLoS One. 2017 Dec 12;12(12):e0188599. doi: 10.1371/journal.pone.0188599 (PMC5726713; doi:10.1371/journal.pone.0188599)
Supplement: S2 Fig — Cellwall represents the 200 x 200 pixelated simulation space (which also can be indexed from 1 to 40,000 using one-dimensional indices). Boundary collects the (orthogonal) boundary pixels of existing glucan exposure sites within the masked stripes. Masked keeps track of which pixels are available to be chosen, however, only those in non-fixed regions cover masked glucan and so can be exposed. The actual MATLAB algorithm was written in such a way that it can be run on multiple processors simultaneously. (DOCX) [file pone.0188599.s002.docx]

**S2 Fig**

$$D_{y}=\frac{y_{experimental}-y_{simulated}}{y_{experimental}}$$

$$D=\left| D_{singletfract} \right|+\left| D_{radii} \right|+\left| D_{density} \right|$$

simspace = 200 % number of pixels along one-dimension of the simulation space

trials = 100 % number of trials to perform for gathering statistics

% Fraction of iterations over which unmasking occurs along boundaries of

% existing glucan exposure sites (if possible):

Pedge = number in the interval [0, 1]

Define fixed (non-striped) exclusion regions.

Masked = (1 : simspace^2) excluding fixed (non-striped) regions

**FOR** i = 1 to trials

% Initialize the pixels in the simulation space to no glucan exposed.

Cellwall = **FALSE**(simspace, simspace)

Boundary = [] % boundary pixels of all glucan exposure sites

**WHIL**E numel(Masked) > 0 % while there are still unmasked pixels ...

rand_index = random element of Masked

% The random unmasking model corresponds to Pedge = 0.

**IF** numel(Boundary) > 0 **AND** rand < Pedge **THEN**

rand_index = random element of Boundary

**END IF**

% Unmask the pixel given by rand_index if not located in a fixed region.

**IF** ~ismember(Masked(rand_index), fixed) **THEN**

Cellwall(Masked(rand_index) = **TRUE**

**END IF**

**IF** Pedge > 0

boundary_nbrs = orthogonal boundary neighbors of Masked(rand_index)

Boundary = [Boundary, boundary_nbrs]

Remove non-unique, previously unmasked pixels and pixels from fixed regions.

**END IF**

Masked(rand_index) = [] % delete this pixel

Collect statistics for this simulation trial.

**END WHILE**


**END FOR**
